# Seed Priming with Jasmonic Acid Counteracts Root Knot Nematode Infection in Tomato by Modulating the Activity and Expression of Antioxidative Enzymes

**DOI:** 10.3390/biom10010098

**Published:** 2020-01-07

**Authors:** Shagun Bali, Parminder Kaur, Vijay Lakshmi Jamwal, Sumit G. Gandhi, Anket Sharma, Puja Ohri, Renu Bhardwaj, Mohammad Ajmal Ali, Parvaiz Ahmad

**Affiliations:** 1 Department of Botanical and Environmental Sciences, Guru Nanak Dev University, Amritsar 143005, Punjab, India; shagun88bali@gmail.com (S.B.); parminder.kaur@gmail.com (P.K.); renubhardwaj82@gmail.com (R.B.); 2 Indian Institute of Integrative Medicine (CSIR-IIIM), Council of Scientific and Industrial Research, Canal Road, Jammu 180 001, India; jamwalvl@rediffmail.com (V.L.J.); sumitggandhi@gmail.com (S.G.G.); 3 State Key Laboratory of Subtropical Silviculture, Zhejiang A&F University, Hangzhou 311300, China; anketsharma@gmail.com; 4 Department of Zoology, Guru Nanak Dev University, Amritsar 143005, Punjab, India; ohri11puja@gmail.com; 5 Department of Botany and Microbiology, College of Science, King Saud University, Riyadh 11451, Saudi Arabia; 6 Department of Botany, S.P. College Srinagar, Jammu and Kashmir 190001, India

**Keywords:** tomato, Jasmonic acid, root knot nematode, oxidative stress, antioxidant enzyme activity, amino acid profiling

## Abstract

The environmental stress, biotic as well as abiotic, is the main cause of decreased growth and crop production. One of the stress-causing agents in plants are parasitic nematodes responsible for crop loss. Jasmonic acid (JA) is recognized as one of signaling molecules in defense-related responses in plants, however, its role under nematode infestation is unclear. Therefore, the present study was planned to traverse the role of JA in boosting the activities of antioxidative enzymes in tomato seedlings during nematode inoculation. Application of JA declined oxidative damage by decreasing O_2_^•−^ content, nuclear and membrane damage under nematode stress. JA treatment elevated the activities of SOD, POD, CAT, APOX, DHAR, GPOX, GR, and PPO in nematode-infested seedlings. Seed soaking treatment of JA upregulated the expression of *SOD, POD, CAT*, and *GPOX* under nematode stress. Various amino acids were found in tomato seedlings and higher content of aspartic acid, histidine, asparagine, glutamine, glutamic acid, glycine, threonine, lysine, arginine, B-alanine, GABA, phenylalanine, proline, and ornithine was observed in seeds soaked with JA (100 nM) treatment during nematode inoculation. The results suggest an indispensable role of JA in basal defense response in plants during nematode stress.

## 1. Introduction

The crop production and food safety are at risk due to the environmental fluctuations, and this environmental change also disturbs the vegetation and ecological balance. Plant parasitic nematodes affect a broad spectrum of vegetable crops and consequently reduce the yield and quality of the crops. Root-knot nematodes (RKN) are placed at the topmost rank in the list of plant pathogens [1]. It is estimated that RKN can cause damage of about US$ 157 billion per annum to worldwide agricultural crops [2]. Nematode infection causes a subsequent decrease in crop yield and made them susceptible to other pathogens too. *Meloidogyne incognita*, has been recognized as the most damaging parasite among RKNs that infect a wide array of crops worldwide. *M. incognita* infests vegetable crops and cause severe loss in the productivity of these crops. Second stage (J_2_) juveniles of these RKNs are the most active and infective ones. They enter the root system by damaging epidermal cells of root tips. They move intercellularly within the vascular cylinder and form a feeding site in the elongation zone of roots. These feeding sites are known as giant cells. The tissue surrounding these giant cells forms particular localized swelling, consequently leading to gall formation. The damaged root system may cause a reduction in water and nutrient uptake and also imbalance the transportation of photosynthates, which results in poor yield and quality of crops [2]. Infection induces alteration in various physiological responses like stunted growth, reduction in chlorophyll content, CO_2_ exchange rate, etc., that are directly affected by the initial concentration of nematodes available in the soil [3,4]. Various reports showed that yellowing of leaves, reduction in plant growth, and weight are the prominent symptoms of RKN infection in tea, sweet basil, and tomato [5,6,7]. An immediate and primary response of plants against pathogen attack is reactive oxygen species (ROS) production that results in hypersensitive responses such as cell death at the infection site. ROS are continuously generated as byproducts of diverse metabolic processes, mainly via electron transport chains in mitochondria and chloroplasts and photorespiration in peroxisomes [8]. They also function as signaling molecules, reported to affect the transcript level of diverse genes and associated with activation and control of different stress-related responses at the molecular level [9,10]. In order to overcome the stress caused by abiotic and biotic factors, plants are equipped with a repertoire of mechanisms to counteract the stress. Antioxidative defense system (enzymatic and non-enzymatic antioxidants) is one such mechanism that plays a pivotal role in stabilizing and evading oxidative damage. ROS-quenching enzymes such as superoxide dismutase (SOD), catalase (CAT), ascorbate peroxidase (APOX), peroxidase (POD), dehydroascorbate reductase (DHAR), glutathione reductase (GR), glutathione peroxidase (GPOX), glutathione-S-transferase activity (GST), etc., are the crucial components of the antioxidative defense system.

Among diverse metabolites, plant hormones mediate essential aspects of growth and developmental activities in plants and also boost up the antioxidative defense system under stress conditions. Jasmonic acid (JA) is an imperative member of the oxylipin family, which plays a decisive role in prompting the systemic resistance response during wounding or pathogen attack [11]. Jasmonates like JA and MeJA are reported to stimulate tolerance during RKN infestation in various plants such as rice, tomato, and *Arabidopsis* [12,13,14]. JA treatment can also decline the number of flea beetles, thrips, and aphids in tomato plants, which, therefore, increase the activities of polyphenoloxidase and proteinase inhibitor [15]. It has been reported that treatment of JA and sodium nitroprusside significantly reduced the number of egg masses and partially improved the net photosynthetic rate and fresh weight in RKN infected tomato plants [16]. These molecules also reduced membrane peroxidation and root electrolyte leakage caused by RKN in tomato plants. RKNs cause significant damage to a wide range of economically important crops, including tomato. Because of high nutritional value, tomato is consumed throughout the world. The use of chemical nematicides is one of the principal approaches to control RKNs, but the usage of these chemical substances may have a detrimental effect on the environment. In the current scenario, scientists are continuously working on the discovery of eco-friendly products to control against RKNs. Our focus is on finding some eco-friendly strategies for the management of the RKNs population. In a quest for exploring an eco-friendly approach and the stress-ameliorative property of JA, the present study was raised to evaluate the role of JA against nematode stress in tomato seedlings by assessing the activity and expression of ROS-scavenging enzymes.

## 2. Materials and Methods 

The nematode culture was maintained in a glasshouse by gathering infected roots from the field. The identification of *M. incognita* was done as described by Chitwood [17]. Egg masses were separated from nematode-infested roots and kept in the Petri plate containing distilled water. Egg masses were kept in the incubator at 26 °C for hatching. Second stage juveniles were collected in the Petri plate containing distilled water, and the number of juveniles was quantified under a light microscope. Tomato cultivar Pusa Ruby, nematode-susceptible variety was used in the present study. Tomato seeds were dipped in different concentrations of JA (0.01, 1, and 100 nM) for 4 h. Thirty seeds were raised in autoclaved petri plates contained Whatman No. 1 filter paper with double-distilled water in it. The Petri plates with seeds were kept in a seed germinator (22–25 °C, 16-h photoperiod, white fluorescent light (intensity 175 μmol m^−2^ s^−1^) and relative humidity of about 80–90%). Inoculation with second-stage juveniles of *M. incognita* (150 juveniles/Petri plate) was done after ideal germination. Seedlings were harvested after seven days of nematode infestation.

### 2.1. O_2_^•−^ Content

The content of O_2_^•−^ was assessed by the method proposed by Wu, et al. [18]. Fresh plant material of one gram was homogenized in 6 mL of phosphate buffer (65 mM, pH 7.8). Homogenates were centrifuged at 12,000× *g* (15 min). The supernatant (0.5 mL) was collected and 0.5 mL of phosphate buffer and 0.1 mL of 10 mM hydrochloride in it. The above reaction was incubated for 30 min at 25 °C. To the above mixture, added 58 mM 3-aminobenzene sulfonic acid (1 mL) and 7 mM 1-napthylamine (1 mL) and again incubated for 20 min at 25 °C. Sodium nitrite was used as a standard for the estimation of O_2_^•−^ content.

### 2.2. Membrane Damage

Membrane damage of plant samples was examined according to the protocol of Gutierrez-Alcala, et al. [19]. The root sections were kept in 50 µM propidium iodide (Sigma-Aldrich, St. Louis, MO, USA) and then incubated for 15 min under dark conditions. Samples were washed with distilled water and mounted on glass slides. Samples were instantly examined under Nikon A1R confocal laser scanning microscope. Excitation (543 nm) and emission (617 nm) wavelengths were used for propidium iodide.

### 2.3. Nuclear Damage

The method of Callard, et al. [20] was used for assessing nuclear damage. Root sections were placed in 4,6-diamino-2-phenylindole (0.1 mg in 100 mL phosphate buffer saline). Sections were kept under dark conditions for 30 min followed by washing with phosphate buffer saline. Root sections were mounted on glass slides and immediately examined under Nikon A1R confocal laser scanning microscope at an excitation wavelength of 358 nm and an emission wavelength of 461 nm.

### 2.4. Protein Content and Antioxidative Enzymes

One gram of fresh plant material was homogenized in 50 mM potassium phosphate buffer (3 mL) under chilled conditions followed by centrifugation at 12,000× *g* at 4 °C for 20 min. The supernatant was collected and stored at −20 °C that was further used for the estimation of protein content. For the estimation of (POD, CAT, APOX, DHAR, and GPOX) activities, plant material was crushed in 50 mM phosphate buffer of pH 7.0 and for GR activity, 50 mM phosphate buffer of pH 7.8 was used. For determination of SOD, plant material was ground well in 50 mM sodium carbonate (3 mL) buffer of pH 10.2. For GST, plant material was homogenized in potassium phosphate buffer (100 mM) of pH 7.5 and for PPO, potassium phosphate buffer (100 mM) of pH 6.0 was used. Protein content was determined according to the protocol of Lowry, et al. [21]. We added 0.9 mL of distilled water to 0.1 mL of plant extract and volume was made up to 1 mL. A test tube with distilled water (1 mL) was used as blank. Various reagents were used for the estimation of proteins (Reagent A: Sodium carbonate (2%) in 0.1 N sodium hydroxide, Reagent B: Copper sulphate (0.5%) in potassium sodium tartrate (1%), Reagent C: Reagent A (50 mL) and reagent B (1 mL) and Reagent D: Folin–Ciocalteau (FC) reagent). Reagent C (5 mL) was added to each test tube followed by shaking and enabled to stand for 10 min. Reagent D (500 µL) was added to the reaction mixture and shaken well. Incubation was done in the dark for 30 min at room temperature. The blue color was generated, and absorbance was taken at 550 nm. Bovine serum albumin (BSA) was used as a standard. A graph was plotted for absorbance vs. concentration for standard solutions of protein. Protein content was calculated from the linear regression equation obtained from the graph.

The SOD activity was estimated by the method of Kono [22]. We placed 1630 µL of sodium carbonate (50 mM, pH 10.2), 500 µL of NBT (24 mM), 100 µL of EDTA (0.1 mM) and 100 µL of triton X-100 in a test cuvette. The reaction was started by the addition of 100 µL of hydroxylamine hydrochloride (1 mM). We appended 70 µL of enzyme extract to the reaction mixture after 2 min. Optical density was recorded at 560 nm. One-unit activity (1UA) of SOD activity was defined as 0.001 ΔA_560_ per min and was expressed as a specific activity in units of enzyme activity per mg protein (U/mg protein).

The activity of CAT was determined according to the method of Aebi [23]. In a test cuvette, reaction mixture contained 50 mM phosphate buffer of pH 7.0 (1500 µL), 15 mM hydrogen peroxide (930 µL), and enzyme extract (70 µL). Absorbance was noted at 240 nm (extinction coefficient of 39.4 mM^−1^ cm^−1^). One-unit activity (1UA) of CAT activity was defined as 0.001 ΔA_240_ per min and was expressed as specific activity in units of enzyme activity per mg protein (U/mg protein).

POD activity was estimated by the method of Pütter [24]. In test cuvette, the reaction mixture comprised of 50 mM potassium phosphate buffer of pH 7.0 (2350 µL), 20 mM guaiacol solution (50 µL), 12 mM H_2_O_2_ (30 µL) and enzyme extract (70 µL). Optical density was recorded at 436 nm (extinction coefficient of 26.6 mM^−1^ cm^−1^). One-unit activity (1UA) of POD activity was defined as 0.001 ΔA_436_ per min and was expressed as a specific activity in units of enzyme activity per mg protein (U/mg protein).

APOX activity was determined by the protocol given by Nakano and Asada [25]. The reaction mixture consisted of 50 mM potassium phosphate buffer of pH 7.0 (2130 µL), 0.5 mM ascorbate (200 µL), 1.0 mM H_2_O_2_ (100 µL) and enzyme extract (70 µL). The absorbance was noted at 290 nm (extinction coefficient of 2.8 mM^−1^ cm^−1^). One-unit activity (1UA) of APOX activity was defined as 0.001 ΔA_290_ per min and was expressed as a specific activity in units of enzyme activity per mg protein (U/mg protein).

The protocol given by Dalton, et al. [26] was followed for the determination of DHAR activity. Reaction mixture consisted of 50 mM potassium phosphate buffer of pH 7.0 (1330 µL), 0.2 mM dehydroascorbate (300 µL), 2.5 mM GSH (500 µL) and enzyme extract (70 µL). Absorbance was recorded at 265 nm (extinction coefficient of 14 mM^−1^ cm^−1^). One-unit activity (1UA) of DHAR activity was defined as 0.001 ΔA_265_ per min and was expressed as a specific activity in units of enzyme activity per mg protein (U/mg protein).

Method of Carlberg and Mannervik [27] was followed for the determination of GR activity. In the test cuvette, reaction mixture included 50 mM potassium phosphate buffer of pH 7.8 (1530 µL), 1 mM EDTA (300 µL), 0.1 mM NADPH (300 µL), 1 mM GSSG (300 µL) and enzyme extract (70 µL). Optical density was taken at 340 nm (extinction coefficient of 6.22 mM^−1^ cm^−1^). One-unit activity (1UA) of GR activity was defined as 0.001 ΔA_340_ per min and was expressed as a specific activity in units of enzyme activity per mg protein (U/mg protein).

GPOX activity was assessed by the method described by Flohé and Günzler [28]. Reaction mixture contained 50 mM potassium phosphate of pH 7.0 (1180 µL), 0.5 mM EDTA (250 µL), 1 mM GSH (250 µL), 0.15 mM NADPH (250 µL), 0.15 mM H_2_O_2_ (250 µL) and enzyme extract (70 µL). Absorbance was read at 340 nm (extinction coefficient of 6.22 mM^−1^ cm^−1^). One unit (1UA) of GPOX activity was defined as 0.001 ΔA_340_ per min and was expressed as a specific activity in units of enzyme activity per mg protein (U/mg protein).

The protocol proposed by Habig and Jakoby [29] was used for the estimation of GST activity. In the cuvette, the reaction mixture comprised of 100 mM phosphate buffer of pH 7.5 (1930 µL), 1 mM GSH (250 µL), 1 mM CDNB (250 µL) and enzyme extract (70 µL). Optical density was taken at 340 nm (extinction coefficient of 9.6 mM^−1^ cm^−1^). One-unit activity (1UA) of GST activity was defined as 0.001 ΔA_340_ per min and was expressed as a specific activity in units of enzyme activity per mg protein (U/mg protein).

PPO activity was estimated by the method of Kumar and Khan [30]. Reaction mixture contained 0.1M potassium phosphate buffer of pH 6.0 (1 mL), 0.1M catechol (0.5 mL) and enzyme extract (0.25 mL). Incubation was done for 2 min followed by adding H_2_SO_4_ (0.5 mL). Absorbance was recorded at 495 nm. One-unit activity (1UA) of PPO activity was defined as 0.001 ΔA_495_ per min and was expressed as a specific activity in units of enzyme activity per mg protein (U/mg protein).

### 2.5. Amino Acid Profiling

Amino acid profiling of plant material was assessed by the protocol given by Iriti, et al. [31] with slight alterations using an amino acid analyzer (Shimadzu, Nexera X_2_). The extract was prepared by homogenizing a fresh plant sample of one gram in 5 mL of 80% methanol. The centrifugation was performed at 10,000 × g for 20 min (4 °C). The supernatant (1 mL) was collected and added sulphosalicylic acid (1 mL) in it, and then again reaction mixture was centrifuged at 10,000 × g for 20 min (4 °C). The reaction mixture was filtered by employing syringe filters of 0.22 µm. We injected 1 µL of the reaction mixture into the vials of the instrument for further estimation of amino acids.

### 2.6. Gene Expression Analysis

Total RNA was extracted by utilizing Trizol (Invitrogen, Life Technologies, Carlsbad, CA, USA) and following the instructions of the manufacturer. cDNA synthesis was performed by the procedure proposed by Awasthi, et al. [32]. Primers were designed for qRT-PCR studies by employing Primer3 software [33] (Table 1). Ubiquitin gene was taken as a house-keeping internal reference gene for normalization. The relative expression level was computed by using 2^−ΔΔct^ procedure [34,35].

### 2.7. Statistical Analysis

Data was presented in the form of mean ± SD and analyzed by two-way analysis of variance followed by honestly significant difference (HSD) Tukey’s test. The experiment was performed three times with replications each time for each treatment.

## 3. Results

### 3.1. Root Fresh Weight

Results showed that root fresh weight was enhanced by 32.1% in nematode inoculated tomato seedlings as compared to non-infected seedlings (Table 2). No significant increase was observed in root fresh weight in JA (100 nM) treated seedlings in comparison to the untreated ones. There was no significant effect observed on root fresh weight in the binary treatment of JA (100 nM) and nematodes, as compared to JA (100 nM) treatment alone. Treatment of JA reduced root fresh weight by 18.7% in nematode inoculated seedlings in comparison to infected seedlings alone (Table 2).

### 3.2. O_2_^•−^ Content

**Figure 1:**
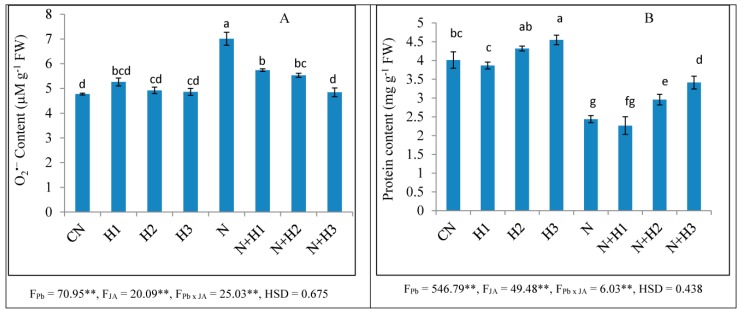
Effect of seed soaking treatment of JA (0.01, 1, and 100 nM) on (**A**) O_2_^•−^ and (**B**) protein contents in tomato seedlings after seven days of nematode inoculation. CN = control, H1 = 0.01 nM JA, H2 = 1 nM JA, and H3 = 100 nM JA. (Mean ± SD, two-way ANOVA, Tukey’s HSD). Means with similar letters are not significantly different from each other. * and ** designated significance at *p* ≤ 0.05 and *p* ≤ 0.01, respectively.

Results indicated increased O_2_^•−^ content by 46.95% in tomato seedlings under nematode infestation in contrast to non-infected seedlings (Figure 1). There was no significant effect observed on O_2_^•−^ content under JA (100 nM) in comparison to untreated seedlings. In binary treatment of JA (100 nM) and nematodes, there was no significant effect was found on O_2_^•−^ content in contrast to JA (100 nM) alone. Application of JA declined the content of O_2_^•−^ in nematode inoculated seedlings. Treatment with 100 nM JA reduced O_2_^•−^ content by 30.94% in nematode infected seedlings in comparison to infested treated seedlings alone (Figure 1).

### 3.3. Membrane and Nuclear Damage

**Figure 2:**
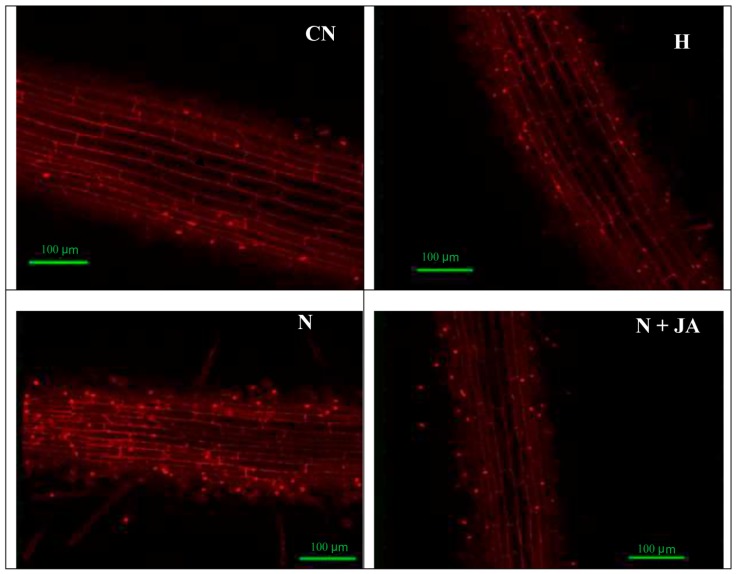
Effect of seed soaking treatment of JA (100 nM) on membrane damage in tomato seedlings after seven days of nematode inoculation. N = nematode, H = 100 nM JA, scale bar = 100 µm.

**Figure 3:**
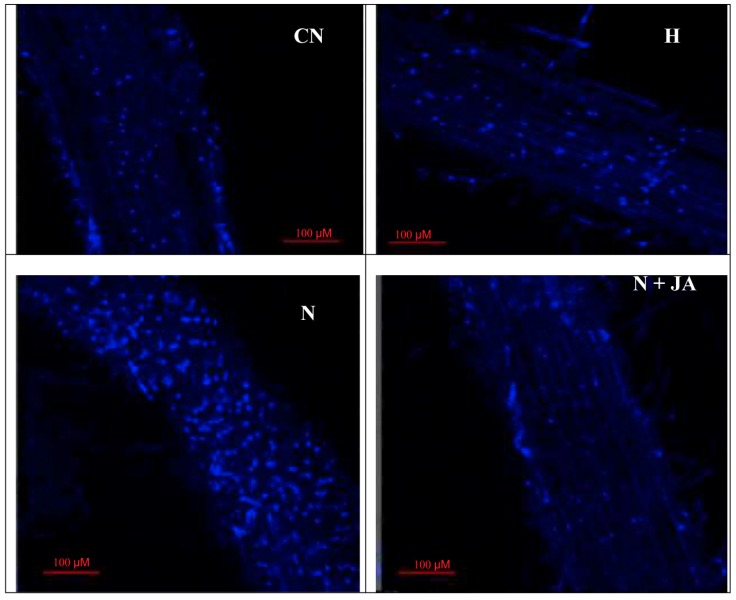
Effect of seed soaking treatment of JA (100 nM) on nuclear damage in tomato seedlings after seven days of nematode inoculation. N = nematode, H = 100 nM JA, scale bar = 100 µm.

Membrane damage due to nematode infection was observed by imaging of tissues under a confocal microscope with propidium iodide, which was indicated by a change in the intensity of red color. High intensity of red color was found in nematode-infested seedlings, which showed more damage in contrast to non-infected seedlings (Figure 2). The intensity of red color declined with JA application. The 100 nM JA treatment alleviated the intensity of red color in nematode inoculated seedlings depicting reduced membrane damage (Figure 2). Similarly, nuclear damage by nematode inoculation was examined by using 4,6 diamino-2-phenylindole (DAPI), which was indicated by a change in the intensity of blue color. High intensity of blue color was recorded in nematode-infested seedlings as compared to non-infected ones (Figure 3). Treatment with 100 nM JA declined the intensity of blue color, which demonstrated the diminution in nuclear damage (Figure 3).

### 3.4. Protein Content and Antioxidative Enzymes Activities

Protein content declined by 46.5% in nematode infected seedlings as compared to uninoculated ones (Figure 1). Protein content was increased by 13.5% in JA (100 nM) treated seedlings as compared to untreated ones. Reduction by 25.1% in protein content was observed in the binary treatment of JA (100 nM) and nematodes as compared to JA (100 nM) alone. Treatment with 100 nM JA increased the protein content by 59.2% in nematode-infested seedlings in contrast to nematode infection alone (Figure 1). Activities of SOD, POD, CAT, APOX, DHAR, GST, GR, and PPO were enhanced by 64.7%, 45.2%, 58.0%, 55.2%, 38.8%, 39.6%, 59.6%, and 86.4%, respectively, in nematode infected seedlings as compared to untreated seedlings (Table 2 and Table 3). Nematode infection declined GPOX activity by 16.7% in tomato seedlings in contrast to untreated ones (Table 3). There was no significant effect observed in the activities of POD, CAT, APOX, DHAR, GST, GR, and PPO in tomato seedlings treated with JA (100 nM) in contrast to untreated seedlings. Activities of SOD, POD, CAT, APOX, DHAR, GPOX, GR, and PPO were increased in binary treatment of JA (100 nM) and nematodes in comparison to JA (100 nM) treatment alone. JA (100 nM) treatment resulted in enhancement in SOD activity (43.0%), POD (48.6%), CAT (52.5%), APOX (29.5%), DHAR (51.8%), GR (70.3%), and PPO (43.8%) in infected seedlings in contrast to nematode infection alone (Table 2 and Table 3).

**Figure 4:**
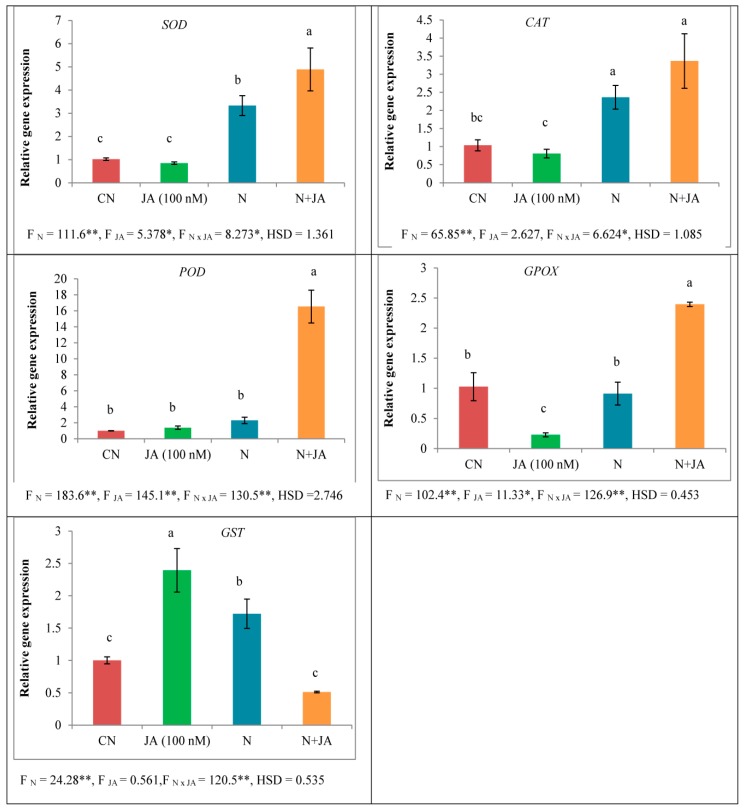
Effect of seed soaking treatment of JA (100 nM) on the expression of antioxidative enzymes (*SOD*, *POD*, *CAT*, *GPOX*, and *GST*) in tomato seedlings after seven days of nematode inoculation. (Mean ± SD, two-way ANOVA, Tukey’s HSD). Means with similar letter are not significantly different from each other. * and ** designated significance at *p* ≤ 0.05 and *p* ≤ 0.01, respectively.

In response to nematode infection, expression of *SOD* (3.277 folds), *CAT* (2.283 folds), *POD* (2.295 folds), and *GST* (1.720 folds) was significantly found enhanced in tomato seedlings as compared to non-infected ones whereas the expression of *GPOX* was found suppressed by 0.8878 folds (Figure 4). Expression of *SOD*, *CAT*, and *POD* had no significant effect under JA (100 nM) treatment in tomato seedlings in comparison to untreated ones whereas the expression of GST was enhanced. In the binary treatment of JA (100 nM) and nematodes, the expression of *SOD*, *CAT*, *POD*, and *GPOX* were increased in tomato seedlings as compared to JA (100 nM) treatment alone (Figure 4). Treatment of JA (100 nM) significantly elevated the expression of *SOD* by 1.469 folds, *CAT* by 1.425 folds, *POD* by 7.204 folds and *GPOX* by 2.626 folds in nematode inoculated seedlings in contrast to non-treated nematode inoculated seedlings, but the expression of *GST* was down-regulated by 0.296 folds (Figure 4).

### 3.5. Amino Acid Profiling

Various amino acids were detected in tomato seedlings and higher content of aspartic acid, glutamic acid, asparagine, glutamine, histidine, glycine, threonine, arginine, B-alanine, GABA, phenylalanine, lysine, proline, and ornithine was detected in seeds primed with 100 nM JA treatment under nematode infection (Table 4).

## 4. Discussion

RKNs are the destructive endoparasites that parasitize various cultivated crops and cause a serious threat to worldwide food security. Hormones play a major role in local and systemic immune responses in plants which further mediate various signaling networks under stress conditions. Among various plant hormones, JA functions as a potential signaling molecule that affects diverse physiological and developmental processes in stressful environments. Earlier studies suggested that JA plays a vital role in the basal defense system against RKNs in various economical important crops. It has been reported that JA significantly reduced the number of eggs/g roots in tomato plants infected with *Meloidogyne javanica* [36]. A study conducted by Lahari, et al. [37] demonstrated that inhibition of strigolactone biosynthesis led to increased accumulation of JA which further caused a reduction in nematode infection in rice. Guo, et al. [38] reported that the application of JA biosynthesis inhibitor decreased the tolerance provided by Rhg1 (resistance to *Heterodera glycines* 1) against the soybean cyst nematode which indicated that JA might play an essential role in Rhg1-regulated tolerance to soybean cyst nematode. It has been suggested that RKN stimulated the accumulation of JA and JA-isoleucine and also the expression of genes involved in the biosynthetic and signal transduction pathway in tomato [39].

In the present work, root fresh weight was enhanced in nematode-infested seedlings as compared to uninoculated seedlings (Table 2), this might be due to the formation of galls in infected roots. Treatment of JA reduced the root fresh weight in infected roots (Table 2). This might be due to a reduction in gall number in nematode infected roots, as shown in the Supplementary Materials (Figure S1). The oxidative stress was elevated in terms of O_2_^•−^ content as well as enhanced nuclear and membrane damage in nematode infected seedlings in the present work (Figure 1, Figure 2 and Figure 3). Overproduction of ROS causes damage to proteins, nucleic acids and lipids [40,41,42]. It has been observed that oxidative damage was induced by the generation of ROS and lipid peroxidation in eggplant, papaya, jasmine, and sour orange during nematode (*Rotylenchulus reniformis, Meloidogyne incognita*, and *Tylenchulus semipenetrans*) infestation [43]. Seeds primed with JA reduced O_2_^−^·content and nuclear and membrane damage in the present study (Figure 1) which is in agreement with the previous studies where treatment of MeJA reduced MDA, O_2_*^•^*^−^, and H_2_O_2_ levels in Cd-stressed rice plants [44]. The reduction in oxidative damage by JA is assumed to be attributed to the enhanced activities of antioxidative enzymes and heme based proteins, which may help in quenching of ROS under abiotic stress [45]. A decrease in protein content and changes in the activities of antioxidative enzymes due to nematode infestation have been observed in the present study which was also earlier reported in green gram plants [46]. Protein content has also been found to be reduced in mung bean plants after 30 days of nematode inoculation [4]. Decline in protein content is attributed to giant cells formation in roots, which lead to gall formation. These giant cells feed upon amino acids for nutrition and decrease their accessibility for protein synthesis. Amino acids are produced by proteolysis of prevailing tissue proteins that lead to a reduction in overall protein content [47].

In the present piece of work, activities of SOD, POD, CAT, APOX, DHAR, GST, GR, and PPO were modulated in nematode-infested seedlings as well as under JA treatment (Table 2 and Table 3). Elevation in the activity of SOD under stress conditions might be due to de novo production of enzyme-associated protein [48]. CAT is an important enzyme which is involved in the elimination of toxic peroxides by converting hydrogen peroxide into molecular oxygen and water [49]. POD is recognized as stress enzyme [50] whereas, APOX and GR are crucial constituents of the ascorbate–glutathione cycle which are involved in quenching of hydrogen peroxide to maintain the redox balance of the cell [51]. APOX employs ascorbate as a substrate for the removal of hydrogen peroxide. GR utilizes NADPH for the reduction of GSSG to GSH and plays an essential role in defense-related responses against stress [52]. SOD catalyzes O_2_^•−^ generated in diverse cell organelles [53]. Under biotic stress, guaiacol peroxide leads to the decomposition of indole-3-acetic acid and is associated with lignin synthesis, which consequently provides resistance to the cells by reducing H_2_O_2_ [10]. DHAR regenerates ASH (reduced form) from oxidized state and mediates the concentration of ASH in the cells which plays an indispensable role against stress. GPOX utilizes GSH for the reduction of H_2_O_2_ and lipid hydroperoxides and protects the plant cells against oxidative damage [54].

It has been found that activities of peroxidase and SOD enhanced in resistant wheat lines (H-93-8) in comparison to susceptible lines of wheat under *Heterodera avenae* (Cyst nematode) infection [55]. Similarly, activities of POD, CAT, PPO, and SOD were increased in the resistant cultivars as compared to susceptible cultivars of tomato after *M. javanica* infection [56]. Another study conducted by Kathiresan and Mehta [57] in sugarcane clones (resistant and susceptible) showed enhanced SOD activity in the leaves and roots of nematode (*P. zeae*) infested clones in comparison to non-infested clones. A study conducted by Xu, et al. [58] observed the effect of *M. incognita* infection on defense-related enzyme activities in resistant (*Solanum torvum*) and susceptible (*S. intergriflium*) varieties of eggplant. Activities of SOD and CAT were higher in resistant cultivar as compared to the susceptible one, whereas the opposite effect was observed in POD activity in resistant and susceptible cultivars. Activities of POD and PPO were enhanced in susceptible as well as resistant varieties of tomato, but the activity was more prominent in resistant varieties [59]. It was reported that SOD, CAT, and APOX activities enhanced under nematode infection in various host plants (eggplant, jasmine, cowpea, orange, and papaya) [43]. Results obtained by the study of Kesba and El-Beltagi [60] demonstrated that nematode-infested grape roots showed an increase in the activities of SOD, APOX, and PPO in comparison to non-infested grape rootstocks. Activities of SOD and CAT were also enhanced in nematode infected tomato seedlings [61]. JA application enhanced protein content in *Glycine max* plants under abiotic stress [62]. JA regulated the synthesis of stress-related proteins which are involved in developmental processes under adverse conditions [63]. It has been elucidated that MeJA has stress ameliorative properties under abiotic stress in soybean plants. Results showed that MeJA improved the activities of SOD, CAT, and POD in stressed plants [64]. Treatment of JA elevated the activities and transcript levels of SOD, CAT, POD, APOX, and PPO in *Brassica napus* under abiotic stress [45]. JA enhanced the activities of CAT, APOX, GST, and GR in *Nicotiana tabacum* [65]. Abiotic stress activated the accumulation of JA which further regulated AsA-GSH pathway by increasing the activities and transcript levels of APOX, MDHAR, DHAR, and GR and contents of GSH and AsA thus played a critical role in tolerance against stress in *Agropyron cristatum* [66]. Alterations in the activities of ROS-scavenging enzymes might be attributed to de novo production or induction of transcription factors associated with defense-related responses under JA application [67].

In the present study, various amino acids were detected in nematode inoculated, as well as JA treated seedlings (Table 4). Under stress conditions, plants accumulate a wide range of metabolites, principally amino acids. In plants, they play a major role in physiological and development processes and also in defense-related responses. Proline, an amino acid, plays a significant role in stressed conditions. It acts as a potential osmoprotectant, signaling molecule and an antioxidant. It maintains osmotic balance, membrane permeability, and generation of ROS under stress [68]. Cysteine is associated with the synthesis of vitamins, methionine, and diverse cofactors and also linked to osmotic balance in plants against stress [69]. Histidine, valine, aspartic acid, and isoleucine are involved in osmoregulation under stress conditions in plant cells [70,71]. Amino acid conjugates of JA may play an imperative role in regulating defense responses in plants under stress [72].

## 5. Conclusions

Results attained from the present study show the stress-protective properties of JA against nematode infestation. Priming of seeds with JA modulated the expression and activities of ROS-scavenging enzymes in nematode-infested seedlings. JA application declined nuclear and membrane damage in nematode inoculated seedlings. Further studies are required to understand the mechanisms behind defense-related responses in plants to endure under stress conditions and also explore fascinating channels to find a connection between physiological and molecular approaches. An earlier study conducted by Bali, et al. [73] and the results obtained from the present study suggested that JA can be used as a potential candidate in biological control of *M. incognita* which can be an efficient approach to lower the usage of chemical nematicides and assist in developing sustainable agriculture practice. Seed priming with JA provides a novel strategy to improve growth and antioxidative defense mechanism under nematode stress and also act as a biocontrol against RKN.

## Figures and Tables

**Table 1:** Primers used for qRT-PCR analysis in the present work.

Gene Name	Primer Sequence
*Ubiquitin*	Forward primer 5′ GAGGAATGCAGATCTTCGTG 3′ Reverse primer 5′ TCCTTGTCCTGGATCTTAGC 3′
*SOD (Superoxide dismutase)*	Forward primer 5′ CAAGATGATGATGGTCCAAC 3′ Reverse primer 5′ CTCCATGTGTCAATTTATTCGG 3′
*POD (Guaiacol peroxidase)*	Forward primer 5′ TGCCCAATGTCGTGTATTC 3′ Reverse primer 5′ CATCAGATGTGGTTGGGT 3′
*CAT (Catalase)*	Forward primer 5′ ACATGGTCCATGCTCTG 3′ Reverse primer 5′ CCCGTCCATATGCCTGTA 3′
*GPOX (Glutathione peroxidase)*	Forward primer 5′ GAGATAATATTCAGTGGAATTTCGCTAA 3′ Reverse primer 5′ GTTGAGGGCTCAACCTT 3′
*GST (Glutathione-S-transferase)*	Forward primer 5′ CATTTGTTATGAATTTATTGAGCAAGAT 3′ Reverse primer 5′ TAAGTGGCCATGTTTCTTCAATATAC 3′

**Table 2:** Effect of seed soaking treatment of JA (0.01, 1 and 100 nM) on root fresh weight and the activities of SOD, POD, CAT, and APOX in tomato seedlings after seven days of nematode inoculation.

Treatments		Root Fresh Weight (g)	SOD (Unit Activity mg^−1^ Protein)	POD (Unit Activity mg^−1^ Protein)	CAT (Unit Activity mg^−1^ Rotein)	APOX (Unit Activity mg^−1^ Protein)
N (Nematode)	JA (nM)					
0	0	0.137 ± 0.003^ef^	14.54 ± 1.39^d^	34.79 ± 3.37^e^	10.51 ± 1.40^de^	31.24 ± 0.62^cd^
0	0.01	0.125 ± 0.004^g^	14.60 ± 2.09^cd^	35.35 ± 2.843^e^	7.86 ± 0.09^e^	27.83 ± 1.56^d^
0	1	0.133 ± 0.004^f^	11.48 ± 1.55^de^	41.15 ± 1.63^de^	8.72 ± 0.75^e^	28.0 ± 1.67^d^
0	100	0.141 ± 0.002^de^	8.23 ± 1.16^e^	43.79 ± 0.75^de^	9.82 ± 0.29^e^	29.67 ± 1.16^d^
N	0	0.181 ± 0.003^a^	23.95 ± 3.24^b^	50.53 ± 228^cd^	16.61 ± 2.55^c^	48.51 ± 4.04^b^
N	0.01	0.165 ± 0.003^b^	28.58 ± 3.31^ab^	58.31 ± 3.01^bc^	19.02 ± 2.02^bc^	49.21 ± 4.31^b^
N	1	0.156 ± 0.002^c^	29.61 ± 0.248^ab^	66.94 ± 4.23^ab^	23.34 ± 1.48^ab^	51.49 ± 1.23^b^
N	100	0.147 ± 0.002^d^	34.25 ± 3.41^a^	75.11 ± 5.32^a^	25.33 ± 2.12^a^	62.82 ± 3.88^a^
F_N_		615.6 **	370.6 **	298.1 **	295.5 **	458.5 **
F_JA_		40.3 **	1.39	28.97 **	8.48 **	9.39 **
F_N×JA_		56.4 **	16.17 **	5.46 **	9.58 **	8.26 **
HSD		0.007	6.077	9.614	4.773	7.781

(Mean ± SD, two-way ANOVA, Tukey’s HSD). Means with similar letter are not significantly different from each other. * and ** designated significance at *p* ≤ 0.05 and *p* ≤ 0.01, respectively.

**Table 3:** Effect of seed soaking treatment of JA (0.01, 1 and 100 nM) on the activities of DHAR, GPOX, GST, GR, and PPO in tomato seedlings after seven days of nematode inoculation.

Treatments		DHAR (Unit Activity mg^−1^ Protein)	GPOX (Unit Activity mg^−1^ Protein)	GST (Unit Activity mg^−1^ Protein)	GR (Unit Activity mg^−1^ Protein)	PPO (Unit Activity mg^−1^ Protein)
N (Nematode)	JA (nM)					
0	0	16.98 ± 2.93^d^	24.10 ± 1.70^abc^	18.93 ± 0.89^b^	11.31 ± 2.23^d^	8.30 ± 1.49^cd^
0	0.01	20.44 ± 0.24^cd^	18.32 ± 0.44^cde^	19.22 ± 1.32^b^	9.14 ± 0.55^d^	6.71 ± 0.11^d^
0	1	20.96 ± 1.96^cd^	16.89 ± 1.05^de^	21.71 ± 1.16^ab^	9.78 ± 0.64^d^	6.86 ± 0.17^d^
0	100	23.08 ± 2.52^bcd^	11.47 ± 0.08^e^	22.37 ± 1.64^ab^	11.94 ± 0.33^cd^	7.76 ± 0.66^d^
N	0	23.58 ± 4.23^bc^	20.07 ± 2.44^bcd^	26.44 ± 3.81^a^	18.06 ± 3.26^bc^	15.49 ± 2.49^b^
N	0.01	28.01 ± 2.79^abc^	20.82 ± 3.64^bcd^	19.96 ± 4.14^b^	20.68 ± 2.36^b^	13.79 ± 3.07^b^
N	1	30.85 ± 1.45^ab^	26.07 ± 0.95^ab^	18.17 ± 2.37^b^	23.16 ± 2.72^b^	16.83 ± 2.01^b^
N	100	35.81 ± 4.08^a^	28.39 ± 4.72^a^	17.05 ± 1.91^b^	30.76 ± 3.15^a^	22.28 ± 1.75^a^
F_N_		63.18 **	37.04 **	0.221	173.5 **	162.58 **
F_JA_		10.81 **	1.42	5.30 **	10.47 **	6.917 **
F_N×JA_		1.39	19.79 **	14.44 **	6.78 **	5.26 *
HSD		8.032	6.996	6.146	6.640	5.266

(Mean ± SD, two-way ANOVA, Tukey’s HSD). Means with similar letter are not significantly different from each other. * and ** designated significance at *p* ≤ 0.05 and *p* ≤ 0.01, respectively.

**Table 4:** Effect of seed soaking treatment of JA (100 nM) on amino acid profiling (µg g^−1^ FW) in tomato seedlings after seven days of nematode inoculation.

**Nematode (N)**	0	0	N	N
**JA (nM)**	0	100	0	100
**Aspartic Acid**	12.08 ± 0.1^b^	20.27 ± 2.9^a^	21.32 ± 1.4^a^	26.24 ± 3.5^a^
**Glutamic acid**	34.96 ± 4.6^b^	52.93 ± 3.6^a^	41.70 ± 1.9^b^	55.97 ± 4.4^a^
**Asparagine**	316.7 ± 28.8^b^	355.68 ± 12.3^b^	389.04 ± 67.4^b^	514.5 ± 27.1^a^
**Serine**	11.39 ± 1.4^a^	11.14 ± 1.8^a^	10.85 ± 0.9^a^	14.22 ± 2.3^a^
**Glutamine**	3242.5 ± 63.4^b^	2770.9 ± 92.5^c^	3157.4 ± 69.6^b^	4454.4 ± 55.6^a^
**Histidine**	26.2 ± 1.4^b^	23.96 ± 4.6^b^	19.58 ± 1.9^b^	35.61 ± 4.9^a^
**Glycine**	2.38 ± 0.3^b^	2.02 ± 0.1^b^	2.00 ± 0.1^b^	3.29 ± 0.1^a^
**Threonine**	21.06 ± 1.2^b^	21.34 ± 2.0^b^	22.19 ± 2.4^b^	30.66 ± 4.2^a^
**Arginine**	136.97 ± 9.5^b^	4.61 ± 0.4^c^	110.22 ± 10.3^b^	215.88 ± 26.4^a^
**B-Alanine**	25.91 ± 4.4^c^	141.03 ± 2.7^a^	32.86 ± 3.1^c^	43.15 ± 3.6^b^
**Alanine**		29.25 ± 2.9^a^		2.75 ± 0.6^b^
**GABA**	4.36 ± 0.2^a^	4.80 ± 2.2^a^	4.60 ± 0.16^a^	5.19 ± 0.4^a^
**Tyrosine**	2.99 ± 0.3	4.46 ± 0.1	4.46 ± 0.14	7.52 ± 0.2
**Cysteine**	13.3 ± 0.4^c^	2.54 ± 0.2^d^	17.25 ± 0.4^a^	16.2 ± 0.3^b^
**Valine**		9.89 ± 0.6	115.69 ± 15.8	46.22 ± 4.8
**Methionine**	242.5 ± 14.2^a^		79.53 ± 5.4^b^	73.99 ± 5.6^b^
**Phenylalanine**	68.21 ± 6.8^c^	85.83 ± 3.5^a^	71.14 ± 1.2^bc^	95.46 ± 5.2^a^
**Isoleucine**	7.16 ± 0.2^b^		6.89 ± 0.7^b^	13.01 ± 1.8^a^
**Leucine**	19.14 ± 1.8^bc^	56.56 ± 4.1^a^	13.20 ± 1.3^c^	21.38 ± 3.2^b^
**Lysine**	69.29 ± 6.8^a^	0.82 ± 0.05^c^	43.09 ± 1.6^b^	69.07 ± 3.8^a^
**Proline**	19.67 ± 2.8^b^	22.29 ± 3.0^b^	22.59 ± 2.9^b^	30.33 ± 3.0^a^
**Ornithine**		21.25 ± 2.9^a^	15.64 ± 1.9^b^	19.98 ± 1.5^ab^

(Mean ± SD, two-way ANOVA, Tukey’s HSD). Means with similar letter are not significantly different from each other (row-wise).

## Supplementary Materials

The following are available online at https://www.mdpi.com/2218-273X/10/1/98/s1.
