# Supplementary material for: Seed Priming with Jasmonic Acid Counteracts Root Knot Nematode Infection in Tomato by Modulating the Activity and Expression of Antioxidative Enzymes
Source: Biomolecules. 2020 Jan 7;10(1):98. doi: 10.3390/biom10010098 (PMC7022828; doi:10.3390/biom10010098)
Supplement: Supplementary file 1 [file biomolecules-10-00098-s001.pdf]

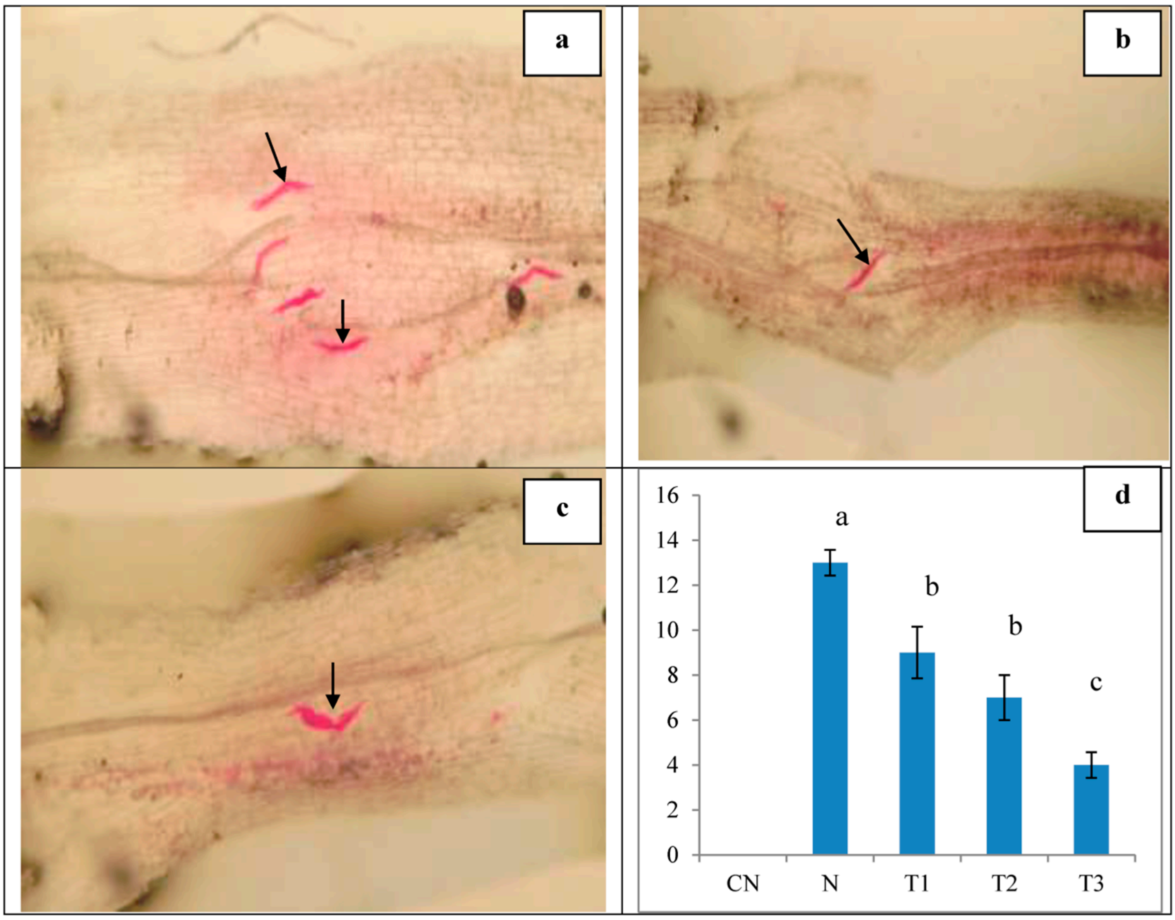

Figure S1. (**a**), (**b**) and (**c**) showed the penetration of nematodes in the roots of tomato (cv. Pusa Ruby) and (**d**) effect of JA on number of galls in the roots of tomato seedlings during nematode infection. CN = Control, N = Nematode, T1 = Nematode + 0.01 nM JA, T2 = Nematode + 1 nM JA and T3 = Nematode + 100 nM JA.
